# Combating Donor Organ Shortage: Organ Care System Prolonging Organ Storage Time and Improving the Outcome of Heart Transplantations

**DOI:** 10.1155/2019/9482797

**Published:** 2019-04-01

**Authors:** Angela Felicia Sunjaya, Anthony Paulo Sunjaya

**Affiliations:** Faculty of Medicine, Tarumanagara University, Jl. Letjen S. Parman No. 1, West Jakarta, Indonesia

## Abstract

**Introduction:**

Cardiovascular diseases are the number one cause of death globally contributing to 37% of all global deaths. A common complication of cardiovascular disease is heart failure, where, in such cases, the only solution would be to conduct a heart transplant. Every 10 minutes a new patient is added to the transplant waiting list. However, a shortage of human donors and the short window of time available to find a correct match and transplant the donors' heart to the recipient means that numerous challenges are faced by the patient even before the operation could be done, reducing their chances of living even further.

**Methods:**

This review aims to evaluate the application of the Organ Care System (OCS^TM^) in improving the efficiency of heart storage based on journal articles obtained from PubMed, Elsevier Clinical Key, and Science Direct.

**Results:**

Studies have shown that OCS is capable of extending the ischemic time 120 minutes longer than conventional methods without any detrimental effect on the recipient nor donor's safety. Based on the PROTECT I and PROCEED II study, 93% of transplantation recipients using the OCS system passed through the 30-day mortality period.

**Discussion:**

OCS is able to prolong the ischemic time of donors' hearts by perfusing the organ at 34°C in a beating state, potentially reducing the detrimental effect of cold storage and providing additional assessment options. Another clear advantage is the implanting surgeon can assess the quality of the donor heart before surgery as well as providing a time safety buffer in unanticipated circumstances that will reduce the mortality risk of transplant recipients.

## 1. Introduction

Cardiovascular disease is currently the number one cause of death globally contributing to 37% of all global deaths [1]. A common endpoint of all cardiovascular disease is heart failure, where, in such cases, cardiac transplantation persists to be the gold standard as it provides excellent long-term survival and a near-normal quality of life [2]. In 2016, 5832 heart transplants were performed globally as reported by the International Society for Heart and Lung Transplantation [3]. However, at the United States alone, at least 4007 people are still at the transplant waiting list. According to the American Heart Association (AHA), from 2001-2008, only 808 out of 1872 cardiac organ donors (43%) were eligible for a heart transplant [4].

In the UK, only 25% of all heart donor candidates are eligible for transplantation. This low percentage is mostly contributed by the donors' age or limited ischemic time. Donor hearts still experience low levels of anaerobic metabolism during cold ischemic time, depleting adenosine triphosphate (ATP) supply and increasing acidosis, marked by elevated lactate levels. Thus with increasing ischemic time, donor hearts are at an increased risk of primary graft failure (PGF). Currently, the maximum ischemic time of 4-6 hours is observed before the heart is classified unsafe for use [5].

Every 10 minutes a new patient is added to the transplant waiting list. Ever increasing demands of heart transplant lists globally coupled with medically constant rates of donor organ procurement results in increased cardiac mortality. On average, 22 people die each day while waiting for a transplant [6]. One of the factors limiting donor heart utilization includes the distance between the donor and recipient hospital. Traditionally, cardiac graft preservation is based on cold ischemic storage. Numerous evidence has found that prolonged cold ischemic time is associated with increased risk of PGF and mortality [2]. The use of an ex vivo normothermic perfusion machine, for example, the Organ Care System (OCS^TM^, TransMedics, Andover, United States), presents a new avenue in expanding the donor pool, reducing cold ischemic time while providing additional donor heart assessment options for clinicians.

## 2. Materials and Methods

This review aims to evaluate the potential application of the Organ Care System (OCS^TM^, TransMedics, Andover, United States) in improving the efficiency of donor heart storage.

### 2.1. Data Source

**Figure 1:**
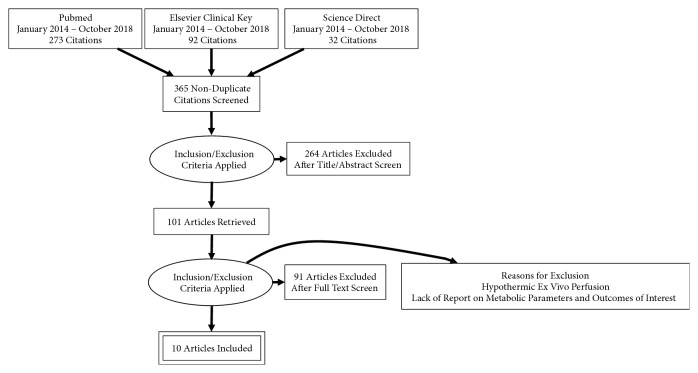
Data selection flow chart.

The databases searched to obtain the articles included PubMed, Elsevier Clinical Key, and Science Direct. The search strategies used included availability of full text and being written in English from the period of January 2014 to October 2018. Keywords used were “*organ care system *or synonyms” OR “ex vivo perfusion” OR “normothermic perfusion” and “*heart transplant*”. When multiple articles of the same study were found, the most recent publication was included. An approach based on title, abstract, and full text was used to evaluate the relevance of articles as shown in Figure 1.

### 2.2. Study Selection

Studies were included if they were original experimental or clinical studies and if they have a randomized controlled trial (RCT), case-control, cohort study, case series, or case studies. In addition, studies were included if (1) they reported the use of OCS on subjects before or after circulatory death, (2) parameters related to cardiac function or length of stay or survival rate or transplant rejection were reported, (3) studies compare OCS use versus routine care (cold storage). The rest were excluded from this review.

## 3. Results

Our initial search resulted in 397 articles found, of which 32 were excluded due to duplicate citations. Two-hundred and sixty four articles were then excluded on the basis of title and abstract; most were studies concerning the use of OCS or ex vivo perfusion not on heart but in lung and renal transplant patients. Of the remaining 101, 91 were excluded because they did not meet the inclusion criteria. Finally, 10 articles were found that fulfilled all of our inclusion criteria, 8 of which were clinical studies with 2 experimental studies – 1 in a porcine model and another in juvenile landrace pigs (Figure 1).

A total of 423 subjects were found from all the studies: 408 from clinical studies and 15 from experimental studies. The majority of studies were performed on standard donor criteria with 2 studies using extended donor criteria. However, there were 3 studies, 1 on humans and 2 on experimental models examining OCS use in heart transplant after circulatory death which is of great interest (Table 1).

## 4. Discussion

**Figure 2:**
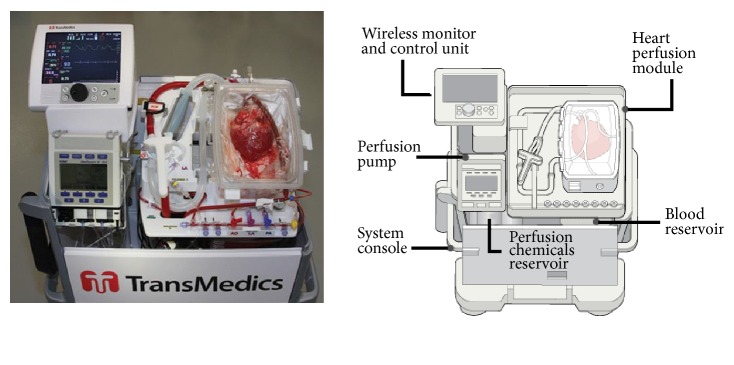
Organ care system with ex vivo perfusion of a human heart [8].

OCS is able to prolong the ischemic time of donors' hearts by perfusing the organ at 34°C in a beating state, potentially reducing the detrimental effect of cold storage and providing additional metabolic (lactate levels) and functional (ejection fraction, cardiac output, and contractility) assessment options. OCS consists of two main components, portable platforms and specific organ perfusion sets, which operate together as one integrated technology [6] (Figure 2).

A heart perfusion platform including oxygen supply and pumps are used to maintain warm, oxygenated, and nutrient-rich blood flow to the heart. Pulsatile flow is maintained with the use of a diaphragmatic pump while a heating plate ensures the maintenance of normothermic conditions. A wireless monitor simultaneously controls the perfusion rate and assists in the evaluation of the donor heart by providing vital information such as aortic pressure, coronary flow rate, temperature, oxygen saturation, and haematocrit levels. The perfusion module is designed to allow for ultrasound assessment and blood sampling for further off-line analysis. The perfusate consists of insulin, methylprednisolone, sodium bicarbonate, antibiotics, multivitamins, and fresh donor blood [17, 18].

### 4.1. Potential Applications of Heart OCS

The use of OCS in heart transplantations is able to potentially reduce cold ischemic time while prolonging cardiac graft preservation. It has been well-documented that prolonged cold ischemia results in greater risks of complications for transplanted patients. OCS use has been found to provide longer ischemic time for donor hearts without adversely affecting patient outcome [8]. One study by Koerner et al. reported lower chances of primary graft failure, episodes of rejection, and cases of acute renal failure in OCS patients (n = 29) compared to cold storage (CS) patients (n = 130) without any significant difference in length of hospital stay, 30-day, 1-year, and 2-year survival rate [13]. The PROCEED II trial (Prospective Multicenter Safety and Effectiveness Evaluation of the Organ Care Device for Heart Use) further supported these findings in which no significant differences were found in Major Adverse Cardiovascular Events (MACE), rejection rates, and length of ICU stay in OCS (n = 63) and CS group (n = 67), respectively (13 vs. 14%; 18 vs. 14%; 147 days vs. 137 days) [16].

Chan et al. reported similar results with no significant differences found in the 2-year survival rate, MACE, and vasculopathy incidence of OCS and CS patients [14]. The longest preservation of a donor heart was reported by Stamp et al. where OCS use was able to extend ischemic time to approximately 10.5 hours before transplantation was finally conducted [19].

With prolonged ischemic time, the donor heart can now travel for longer distances, expanding the list of potential recipients and increasing the chances of gaining a matching donor heart. This reduced ischemic time is especially beneficial in patients with previous cardiac surgery or in redo transplantation, giving the surgeons additional time to safely lyse all the adhesions and prepare the cuffs before arresting the heart in the OCS, thus allowing better prognostic outcomes.

In addition, the urgency and risk associated with ischemic time often results in the need of a dangerous high-speed trip for the transplant team, which increases the risk of serious injury and death on the road. In the United States, 122 accidents and 68 injuries were reported by transplant teams from the period of 1990-2007 in which only 16% of respondents felt safe during the transportation of a donor heart to the recipient hospital [20]. Increasing safety and comfort during the transportation of a donor organ could also be indirectly attributed with the use of OCS.

Another advantage for the ex vivo perfusion of donor hearts is the expansion of the donor pool and acceptance of marginal donors. The inclusion of donor hearts with a high risk of an adverse donor-recipient profile or the so-called “extended criteria” shows great promise in increasing the supply of donor hearts available today [9, 21]. Currently, fewer than 50% of potential donors in the United States become actual organ donors. The extended criteria includes potential donors with ages between 55 and 65, a reduced left ventricular (LV) function (left ventricular ejection fraction > 30 and <50%), coronary one-vessel disease, significant but not detrimental catecholamine support, moderate LV hypertrophy (>13 and <17 mm), and/or distance resulting in an expected warm ischemic time >180 min [7].

A study conducted by Saez et al. evaluating the use of OCS in extended criteria donor hearts (n=26) found a 100% 30-day survival rate, preserved cardiac function in 92% of patients with a mean duration on inotropic support of 113 ± 85 hours, and a length of ICU stay of 6 days [12]. In a subsequent study, OCS use was found to also benefit high-risk recipients such as those with left ventricular assisted devices (LVAD) where prolonged ischemic time is expected. In high risk recipients, OCS use was associated with less blood loss (900 mL vs. 1475 mL), duration of inotropic support (96 hours vs. 204 hours), and length of ICU stay (7 days vs. 25 days) although they were not found to be significant [22].

The use of donated after circulatory death (DCD) donor hearts also present a new avenue in increasing organ supply [23]. Using DCD hearts is projected to increase the number of hearts available for transplantation by more than 10% [10]. However, the retrieval of such organs has been controversial. According to the Maastricht classification, the categories of DCD can be divided into 4 groups: type I includes death on arrival and having not been resuscitated; type II includes being unsuccessfully resuscitated, type III includes typical controlled DCD, with planned cardiac arrest; and type IV includes planned donations following brain death (DBD) that suddenly arrest during or after the brain death determination. Types I, II, and IV are classified as uncontrolled DCD, in which cardiopulmonary resuscitation is typically conducted before any organ recovery procedures can be employed [9].

In an animal study conducted by Saez et al., donor hearts from nonheparinized donation after cardiac death (DCD) porcine donors were found to be successfully resuscitated using the application of OCS [11]. Similar results were obtained by Iyer et al. with the primary endpoint being the ability to wean off cardiopulmonary bypass (CPB) and maintain hemodynamic stability for at least 3 hours after weaning. All OCS hearts are able to be weaned off cardiopulmonary bypass (CBG) and maintain stable hemodynamics 3 hours after weaning whereas none of the 3 hearts in the CS group could be weaned from CPB, despite escalation of inotrope doses. CS donor hearts were unable to establish stable cardiac rhythm and were associated with poor contractility [15]. The results of these findings were soon followed by the 1^st^ successful DCD human heart transplant in St. Vincent's Hospital [24].

The OCS device also enables the implanting surgeon to assess the quality of the donor heart before any irreversible steps are undertaken on the recipient. Therefore, in any unanticipated circumstances, a time safety buffer is available, thus reducing the mortality risk in transplant patients. Alongside a standard hemodynamic assessment of the donor heart, further examination such as echocardiography, lactate profile, serum creatine kinase MB levels, and other examinations for suspected coronary artery disease (CAD) can be done to ensure the quality of donor hearts before transplantation. CAD can be suspected in the presence of persistent aortic pressure unresponsive to increased adenosine infusion or increased lactate profiles (more than 5 mmol/l) [25].

In the OCS PROCEED II trial, 79 donor hearts were included in the study initially out of which 3 were eventually excluded based on lactate profile. A confirmatory histopathological examination of these hearts further revealed significant myocardial contusion in two of the donor hearts and undiagnosed left ventricular hypertrophy in the other. Thus, an elevated lactate profile can potentially serve as an important tool in assessing the viability of donor hearts before any irreversible steps are taken [16].

This study was further confirmed by Tsui et al. in a study at Papworth Hospital and UCLA on the potential assessment capabilities of OCS involving 14 donor hearts. Donor hearts were assessed based on hemodynamic, echocardiography, and lactate profiles and evaluated for the presence of palpable coronary disease. Based on these parameters, 12 donor hearts were eventually accepted for transplantation while 2 were turned down based on end lactate profile despite adequate coronary flow. Further histopathological examination of one of the rejected donor hearts revealed widespread triple vessel coronary artery disease with >95% stenosis. The second donor displayed significant left and right ventricle wall myocardial contusion upon further investigation [26].

Other avenues that could be explored on the ex vivo assessment capability of OCS include intravascular ultrasound and coronary angiography for the detection of occult coronary artery disease as well as contrast echocardiography to ensure myocardial perfusion [27, 28]. The summary of the study results can be found in Table 2.

### 4.2. Limitations of Heart OCS

Additional resources such as support personnel, equipment, appropriate transport, and the collection of donor blood are required for the application of OCS thus making it a more costly endeavour compared to conventional cold storage preservation. NICE estimated a £30,758 cost per transplant by using OCS alone, not taking into account additional costs incurred during the hospital stay whereas cold storage of organs was found to cost only an estimated £118,80 per transplant [29].

However, the long-term benefits of potentially making more donor hearts available for transplantation must be taken into consideration as well as the reduced medical costs incurred with a lower risk primary graft failure (PGF). Forty-three percent of patient deaths in the first 30 days after transplantation are a result of PGF. One of the main reasons for the costliness of heart transplantation includes the fact that when a recipient develops PGF thus requiring additional circulatory support and prolonged intensive care stay. Compared to the potential long-term costs from PGF, both financially and in quality of life, the use of OCS regardless of its cost can be financially justified.

## 5. Conclusion

The use of an ex vivo normothermic perfusion machine, for example, the Organ Care System (OCS^TM^, TransMedics, Andover, United States), presents a new avenue in expanding the donor pool, reducing cold ischemic time while providing additional donor heart assessment options for clinicians. Intercollaborative effort between government, medical institutions, and clinicians as well as the publication of a clear set of guidelines regarding the usage of OCS would be helpful in increasing the number OCS use. Further studies could be done regarding the usage of OCS in an extended donor pool, DCD hearts, evaluation of its donor heart assessment capabilities, and the link between donor lactate levels with outcomes in the future.

## Figures and Tables

**Table 1:** Study Characteristics.

Study Characteristics					
Studies	Year	Subjects	N		Donor Criteria
			Cold Storage	OCS	
Saez et al. [7]	2014	Humans	-	26	Extended^*∗*^

Koerner et al. [8]	2014	Humans	130	29	Standard

Yeter et al. [9]	2014	Humans	-	21	Extended^*∗*^

Saez et al. [10]	2015	Porcine Model	-	5	Donation after circulatory death (DCD)

Iyer et al. [11]	2015	Juvenile Landrace pigs	3	7	Donation after circulatory death (DCD)

Saez et al. [12]	2015	cf-LVAD patients	15	15	Standard

Ardehali et al. [13]	2015	Humans	63	67	Standard

Stamp et al. [14]	2015	Human	-	1	Standard

Dhital et al. [15]	2015	Humans	-	3	Donation after circulatory death (DCD)

Chan et al. [16]	2017	Humans	19	19	Standard

^**∗**^Extended donor criteria - donor hearts with a high risk of an adverse donor-recipient profile.

**Table 2:** Study Results.

Study Results						
Studies	Cold Ischemic Time (min)	OCS Perfusion Time (min)	Total Time (min)	Length of Hospital Stay	30-day survival (%)	Comments
Saez et al. [7]	85 ±17	284 ± 92	371 ± 102	39 ± 29 days	100%	(i) Cardiac function preserved in 92% of patients (ii) Mean duration on inotropic support – 113 ± 85 hours (iii) Length of ICU stay – 6 days (iv) Longest period of OCS support - 464 minutes

Koerner et al. [8]	-			26 days	96%	(i) 30-day survival rate (OCS: 96%; CS: 95%) (ii) 1-year survival rate (OCS: 89%; CS: 81%) (iii) 2-year survival rate (OCS: 89%; CS: 79%) (iv) Lower primary graft failure (OCS: 6.89%; CS: 15.3%) (v) Lower episodes of rejection (OCS: 17.2%; CS: 23%) (vi) Lower cases of acute renal failure (OCS: 10%; CS: 25.3%) (vii) No difference in hospital stay (OCS: 26 days; CS: 28 days)

Yeter et al. [9]	68	320	388	-	95%	(i) Survival rate of 95% at 30 days and 6 months and 87% at one year and four years, respectively. (ii) No post-transplant vasculopathy incidences were reported

Saez et al. [10]	240			-	-	(i) 4 hearts were able to resuscitated (ii) 3 were transplantable with excellent visual contractility and lactate trends (iii) One was found unsuitable for transplantation with abnormal lactate levels and impaired contractility

Iyer et al. [11]	90	240	330	-	-	*OCS Hearts * (i) All OCS hearts are able to be weaned off cardiopulmonary bypass (CBG) and support the animal's circulation with the use of inotropic support (ii) Stable hemodynamics 3 hours post weaning *Cold Storage (CS)* (i) None of the 3 hearts in the CS group could be weaned from CPB, despite escalation of inotrope doses. (ii) Unable to establish stable cardiac rhythm (iii) Poor contractility

Saez et al. [12]	89 ± 17	312	373 ± 95	37.5 days	73.3%	(i) *Cold Storage:* Cold Ischemic Time (204 ± 29 min) (ii) 30-day survival rate (OCS: 73.3%, CS: 100%) (iii) Blood loss within 24h (OCS 900 mL, CS 1475 mL) (iv) Duration of inotropic support (OCS 96 hours, CS 204 hours) (v) Length of ICU stay (OCS 7 days, CS 25 days)

Ardehali et al. [13]	113	211	324	-	94%	(i) *Cold Storage:* Cold & Total Ischemic Time (195 min) 30-day survival rate (97%) (ii) No significant difference in MACE, rejection rates and length of ICU stay in OCS and CS group respectively (13 vs 14%; 18 vs 14%; 147 days vs 137 days)

Stamp et al. [14]	105	503	611	15	100%	No episodes of rejection 1-year post transplant.

Dhital et al. [15]	25 ± 3	257 ± 12	232	91-176 days	100%	1^st^ successful DCD heart transplant in a human

Chan et al. [16]	134 ± 45	-	361 ± 96	-	-	(i) *Cold storage:* Cold & total ischemic time - 207 ± 50 min (ii) No difference in 2-year patient survival rate (OCS: 72.2%; cold storage: 81.6%; p = 0.38) (iii) Allograft rejection (OCS: 53%; cold storage: 61.8%) (iv) No difference in MACE and vasculopathy incidences
